# Modified fructan accumulation through overexpression of wheat fructan biosynthesis pathway fusion genes *Ta1SST:Ta6SFT*

**DOI:** 10.1186/s12870-024-05049-w

**Published:** 2024-04-30

**Authors:** Tong Chen, Matthew Hayes, Zhiqian Liu, Daniel Isenegger, John Mason, German Spangenberg

**Affiliations:** 1 Agriculture Victoria, Agribio, Bundoora, VIC Australia; 2 School of Applied Systems Biology, La Trobe University, Bundoora, VIC Australia; 3 Present Address: Qingdao Agricultural University, College of Grassland Science, N0. 700 Changcheng Road, Chengyang District, Qingdao, Shandong Province 266109 P.R. China

**Keywords:** Fructan, Wheat, Water deficit, Ta1SST, Ta6SFT

## Abstract

**Background:**

Fructans are water-soluble carbohydrates that accumulate in wheat and are thought to contribute to a pool of stored carbon reserves used in grain filling and tolerance to abiotic stress.

**Results:**

In this study, transgenic wheat plants were engineered to overexpress a fusion of two fructan biosynthesis pathway genes, wheat sucrose: sucrose 1-fructosyltransferase (*Ta1SST*) and wheat sucrose: fructan 6-fructosyltransferase (*Ta6SFT*), regulated by a wheat ribulose-1,5-bisphosphate carboxylase/oxygenase small subunit (*TaRbcS*) gene promoter. We have shown that T4 generation transgene-homozygous single-copy events accumulated more fructan polymers in leaf, stem and grain when compared in the same tissues from transgene null lines. Under water-deficit (WD) conditions, transgenic wheat plants showed an increased accumulation of fructan polymers with a high degree of polymerisation (DP) when compared to non-transgenic plants. In wheat grain of a transgenic event, increased deposition of particular fructan polymers such as, DP4 was observed.

**Conclusions:**

This study demonstrated that the tissue-regulated expression of a gene fusion between *Ta1SST* and *Ta6SFT* resulted in modified fructan accumulation in transgenic wheat plants and was influenced by water-deficit stress conditions.

**Supplementary Information:**

The online version contains supplementary material available at 10.1186/s12870-024-05049-w.

## Background

Fructans are linear or branched polymers of fructose and can be found in 12 to 15% of all angiosperm species [1]. In fructan-accumulating plants such as wheat, fructans are mainly stored in stems along with other water-soluble carbohydrates (WSC) [2] which are utilised for grain filling [3, 4] and can contribute to abiotic stress tolerance [5, 6]. Fructan polymers typically consist of one glucose residue and several fructose residues, of which the length, or degree of polymerisation (DP), refers to the number of fructose subunits [7]. 1-kestose, which contains one glucose and two fructose residues, is DP = 3 and is the smallest fructan molecule used as a precursor in the synthesis of longer fructan polymers [6]. Furthermore, longer fructan polymers can be classified into four basic subgroups: (1) Inulin-type fructans; (2) levan-type fructans; (3) graminan-type fructans and (4) neo-type fructans [6]. Both inulin- and levan-type fructans are linear with either β (2,1) linkages or β (2,6) linkages connecting the fructose residues. Graminan-type fructans are branched with both β (2,1) and β (2,6) linkages connecting the fructose residues [6]. Inulin, levan and graminan subgroups share a common structure with one terminal glucose residue and an elongation of fructose residues from position 1 of the glucose unit. However, the neo-type fructans differ and contain one internal glucose residue with elongations of fructose polymers from both position 1 and 6 of the glucose unit [6].

Four types of plant fructan biosynthetic enzymes have been characterised. They are, sucrose: sucrose 1-fructosyltransferase (1-SST) (EC 2.4.1.99) [8], fructan: fructan 1-fructosyltransferase (1-FFT) (EC 2.4.1.100) [9], sucrose:fructan 6-fructosyltransferase (6-SFT) (EC 2.4.1.10) [10] and fructan: fructan 6G-fructosyltransferase (EC 2.4.1.243) (6G-FFT) [11, 12]. In wheat, genes encoding fructosyltransferases, such as *Ta1SST*, *Ta1FFT*, and *Ta6SFT*, have been studied and observed to contribute to cold hardening in winter wheat [13, 14]. During grain development, peaks in fructan accumulation and 1-SST and 6-SFT enzyme activity were reported during the first phase of grain filling when the caryopsis structure is laid down and actual grain-filling is initiated [15]. To date there has been no molecular characterization of a gene encoding a 6G-SFT enzyme, however, the detection of the trisaccharide 6G-kestotriose (neo-kestose) in wheat flour suggests probable 6G-SFT activity during grain development [16]. Furthermore, the *TaMYB13* transcription factor has been shown to have a regulatory function in fructan biosynthesis by binding to the promoters of *Ta1SST* and *Ta6FT* genes in flag leaf and top internode at anthesis [17].

In wheat, WSC stored in stems can contribute up to 30% of the final grain weight at maturity [4], and a positive correlation between the level of fructan accumulation in stems at anthesis and grain yield in wheat has been reported under WD conditions [3, 15, 18–21]. Consequently, high fructan accumulation in vegetative tissues is considered a desirable trait in breeding due to its role in grain yield and abiotic stress tolerance [20, 22–24]. Interestingly, no total yield advantage under stress-free field conditions have been found with high fructan-wheat due to a lower grain count per head and partially compensated for by increased single grain mass [25]. Nevertheless, elucidating the role of fructans and their potential to improve crop yield under drought conditions is of continuing interest [6]. It is thought that fructans have various roles in stress tolerance. Firstly, fructans work as an osmolyte enabling osmotic adjustment which maintains cellular water potential [6]. Secondly, fructans can stabilize cell membranes by forming fructan-polysaccharide complexes [6]. Thirdly, fructans may have antioxidant-like properties by acting as reactive oxygen species (ROS) scavengers [26]. Finally, fructans are associated with stress-induced biochemical cascades such as anthocyanin production and hormone regulation [27, 28].

Indeed, studies with transgenic plants have enabled functional characterisation of isolated fructan genes and reported a potential role for these genes in stress tolerance. Tobacco plants transformed with bacterial levansucrase, which is a bacterial form of 1-SST, showed increased tolerance to drought [29] and cold stress [30]. Overexpression of wheat fructosyltransferase genes *Ta1SST* and *Ta6SFT* enhanced abiotic stress tolerance in naturally fructan-accumulating plant species such as ryegrass [31] and triticale [32], and in non-fructan-accumulating species such as rice [33] and tobacco [34]. Grains produced by transgenic triticale plants modified with seed-specific expression of *Ta1SST* and *Ta6SFT* had 50% lower starch content, 10 to 20 times higher fructan content than non-transgenic controls and showed increased fructan accumulation in germination and under cold stress conditions [32].

In this paper we characterised fructan biosynthesis in wheat plants, that contain a cisgenic gene fusion of the fructan biosynthesis genes, *Ta1SST* and *Ta6SFT*. Selected transformation events were characterised to the T4 generation for transcription and metabolite production and accumulation under well-watered and water-deficit conditions.

## Results

### Transcription of a *Ta1SST:Ta6SFT* fusion in transgenic wheat leaf samples

**Fig. 1:**
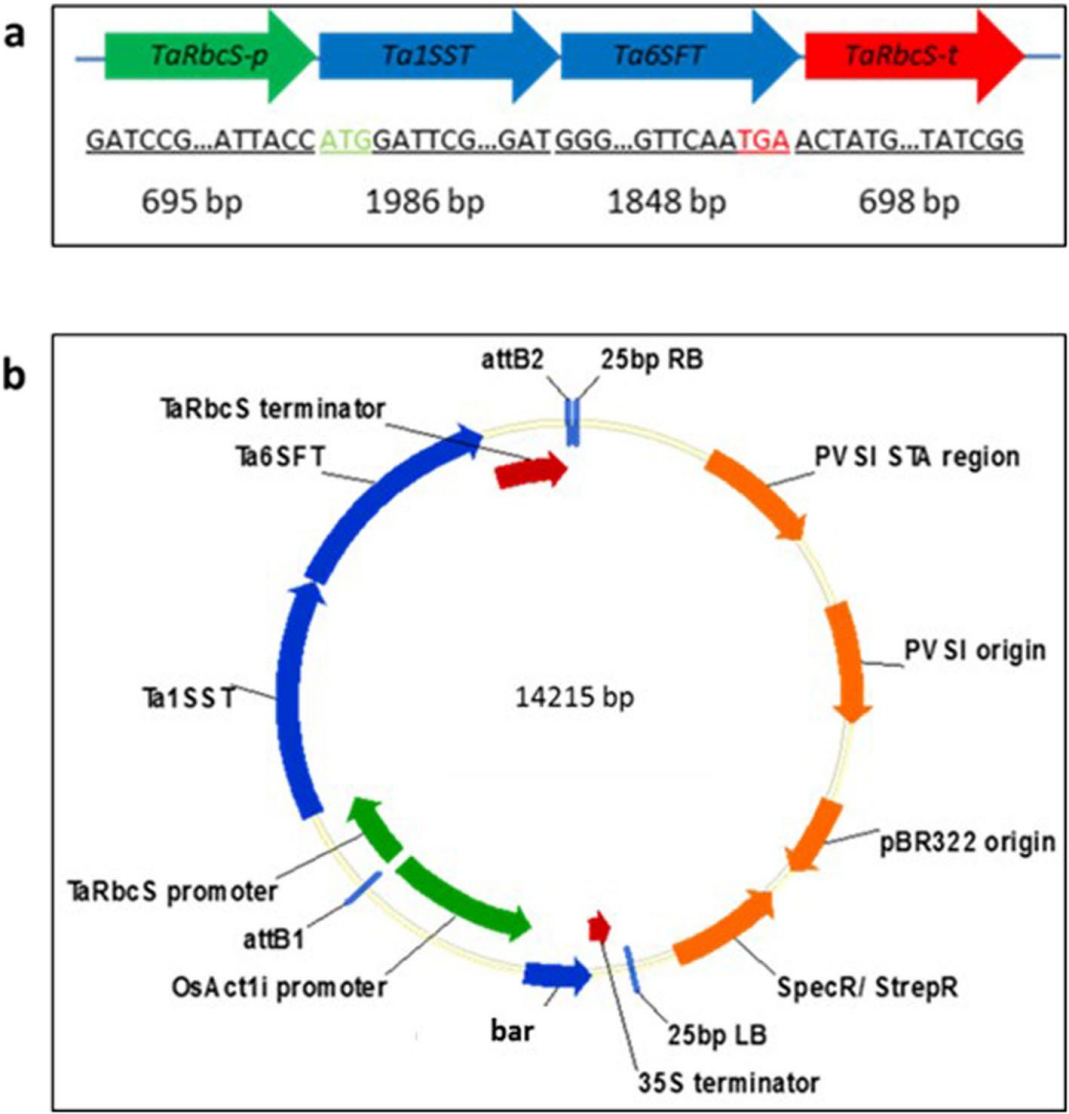
Illustrations of the gene constructs and the binary vector. **a** GOI contains the coding sequence (CDS) of Ta1SST and Ta6SFT, which were fused together as a single transcriptional sequence, and regulated by a TaRbcS promoter and terminator (TaRbcS-p_Ta1SST:Ta6SFT_TaRbcS-t); and (**b**) the vector contains a selection marker (OsActin-p _bar_35S-t) of 14,215 bp. Abbreviations: attB1 and attB2: attachment site B1 and B2 on the E. coli chromosome; 25bp LB and 25bp RB: 25bp left and right border of T-DNA; PVSI origin: pVS1 replication origin from P. aeruginosa plasmid pVS1; PVSI STA region: the stability region from Pseudomonas plasmid pVS1; pBR322 origin: low copy replication and maintenance sequence from E. coli plasmid pBR322; SpecR/StrepR: spectinomycin/ streptomycin resistance gene. The features in illustration (a) are not in scale

**Fig. 2:**
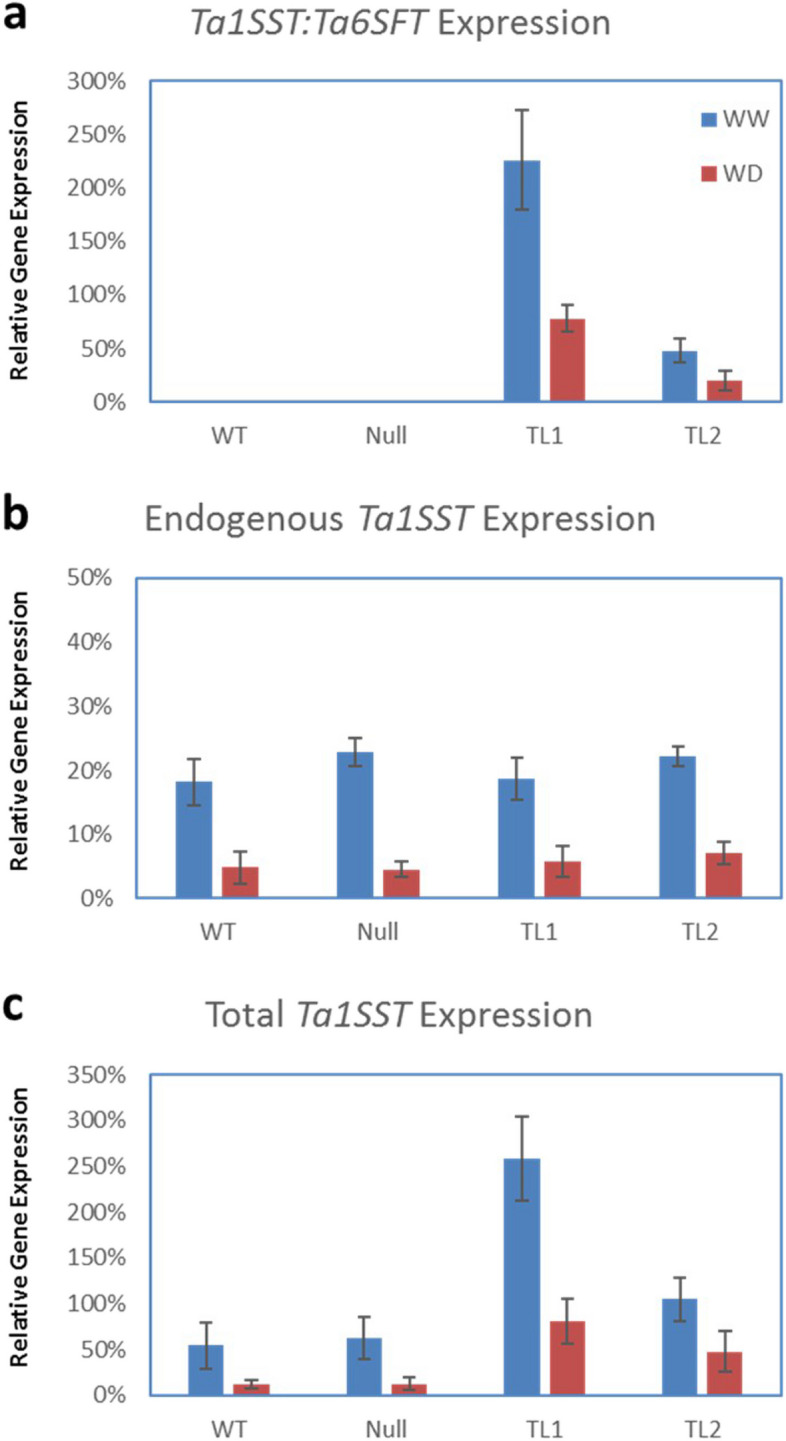
The relative gene expression levels of the (**a**) Ta1SST:Ta6SFT transgene; (**b**) endogenous Ta1SST gene and (**c**) combined Ta1SST:Ta6SFT transgene and endogenous Ta1SST gene. Mature flag leaf samples from WT, null, TL1 and TL2 grown under either WW or WD conditions were used, and relative gene expression levels were normalised against the housekeeping gene Ta2776 [35] in all assays. Average values and standard deviations were calculated from 4 biological replicates of each line under each treatment

Transcription of the fructan biosynthesis transgene *Ta1SST:Ta6SFT* (Fig. 1) was detected in transgenic events (TL1 and TL2), but not in non-transgenic (NT) plants (Fig. 2). After normalisation against an endogenous reference gene *Ta2776* [35], relative transgene expression level in event TL1 was approximately fourfold higher than in TL2 under both well-watered (WW) and WD treatments (Fig. 2a). In both transgenic events, the transgene expression level in WW plants was approximately twofold higher than in WD plants (Fig. 2a). Transgenic and wild type (WT) plants showed similar levels of endogenous *Ta1SST* expression and the WW plants showed approximately fourfold higher *Ta1SST* expression than the WD plants (Fig. 2b). The total *Ta1SST* (transgenic and endogenous) expression levels in events TL1 and TL2 showed approximately fourfold and twofold increases respectively compared to WT and null plants under both WW and WD treatments. Transgenic, WT and null WD plants showed reduced total *Ta1SST* expression compared with WW plants (Fig. 2c). Similar results were also observed when using a different reference gene (*TaGAPDH*) (data not shown).

### Thin Layer Chromatographic (TLC) analysis of vegetative tissues

**Fig. 3:**
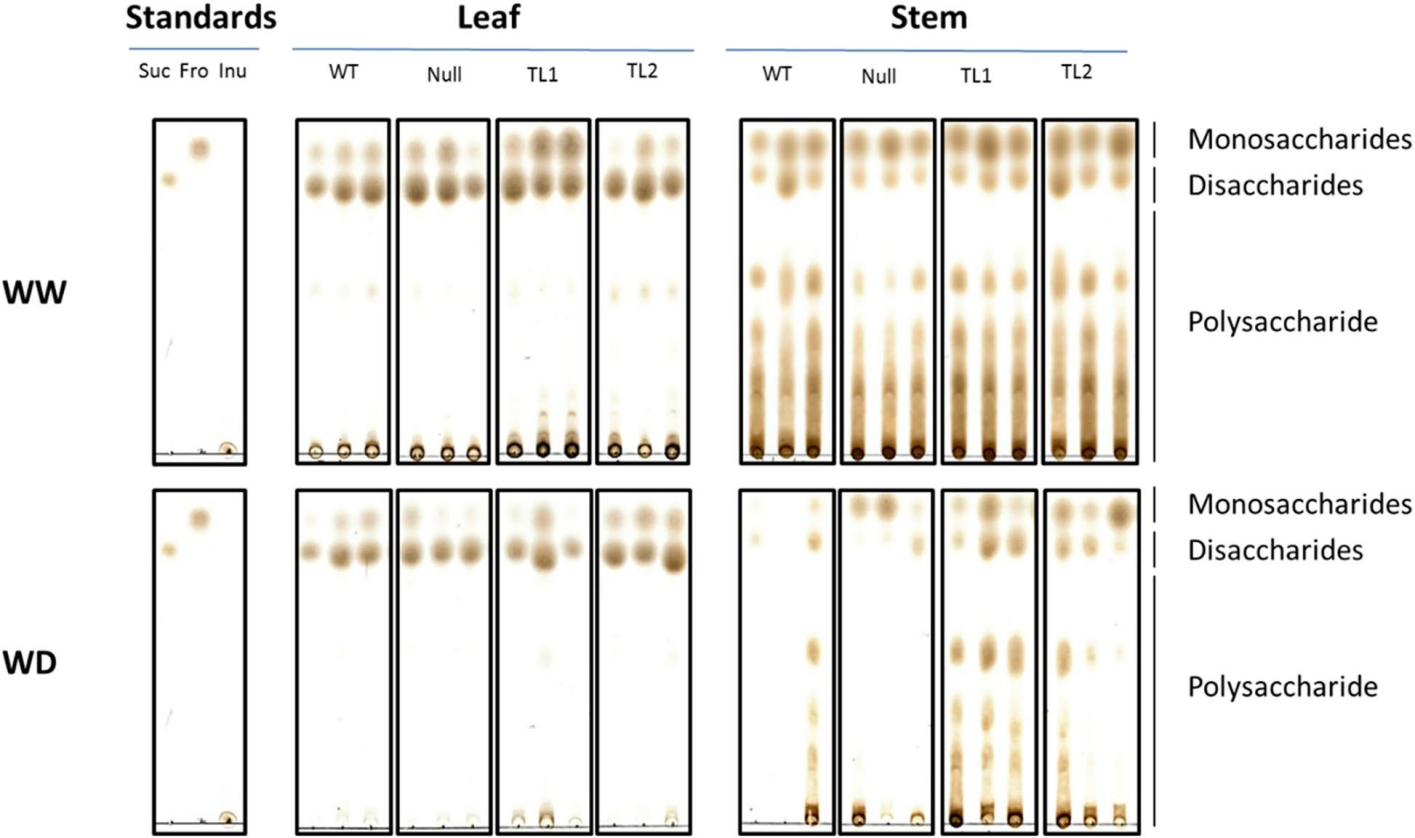
TLC plates depicting the presence and absence of WSC molecules in leaf and stem samples of WT, null, TL1 and TL2 plants under WW and WD conditions. WSC extract, 2 µL, of 3 leaf and 3 stem samples from WT, Null, TL1 and TL2 events under both WW and WD conditions were loaded onto TLC plates and separated based on their molecular weights. Smaller molecules, such as monosaccharides and disaccharides, migrate further to the top of plate. 2 µl of 2 mg/mL Sucrose (Suc), fructose (Fru) and inulin (Inu) were used as controls. TLC plates were scanned, cropped, assembled, and annotated for publication

WW leaf samples from transgenic events TL1 and TL2 showed slightly elevated concentrations of high-DP WSC polymers compared to samples from WT and null plants (Fig. 3). In WW stem samples, no difference could be observed between transgenic and non-transgenic plants (Fig. 3). In WD plants, a slight increase in high-DP polymers was observed in leaf samples of both transgenic lines when compared with non-transgenic lines. Generally, stem samples showed higher concentrations of high-DP WSC polymers, however, biological replicates were not always consistent with non-transgenic samples, especially in WD treated stem samples, which showed a range in concentration from negligible to relatively high. Transgenic lines more consistently contained relatively high concentrations of high-DP WSC polymers (Fig. 3).

### Liquid Chromatography—Mass Spectrophotometry (LC–MS) analysis in leaf, stem and grain

LC–MS showed and validated that fructan polymers (DP3-DP10) from leaf, stem and grain samples of transgenic and WT plants from WW and WD treatments could be separated and identified based on their accurate mass and their relative abundance measured using peak area (Supplementary Fig. 1).

**Fig. 4:**
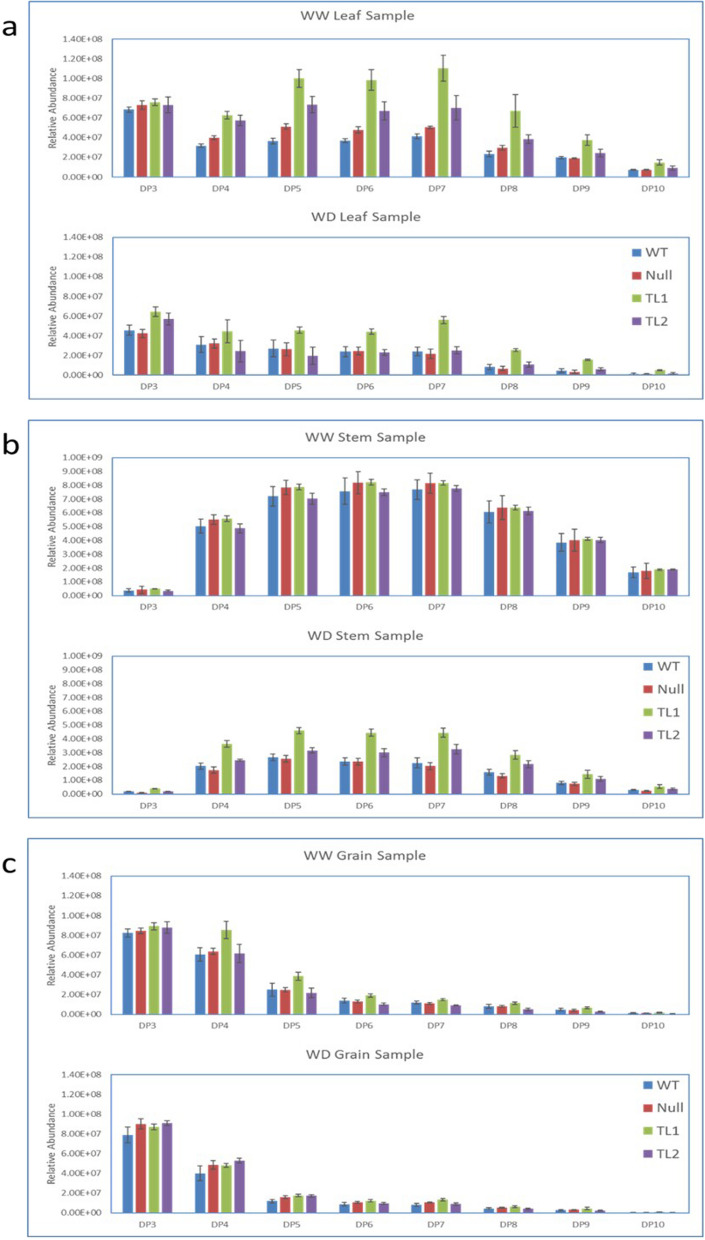
Relative abundance of DP3 to DP10 fructan polymers extracted from (**a**) leaf; (**b**) stem and (**c**) grain samples of WT, null, events TL1 and TL2 plants under WW and WD conditions. Mean values and standard deviations (represented as error bars) were calculated from 4 biological replicate samples of each line of each treatment

**Fig. 5:**
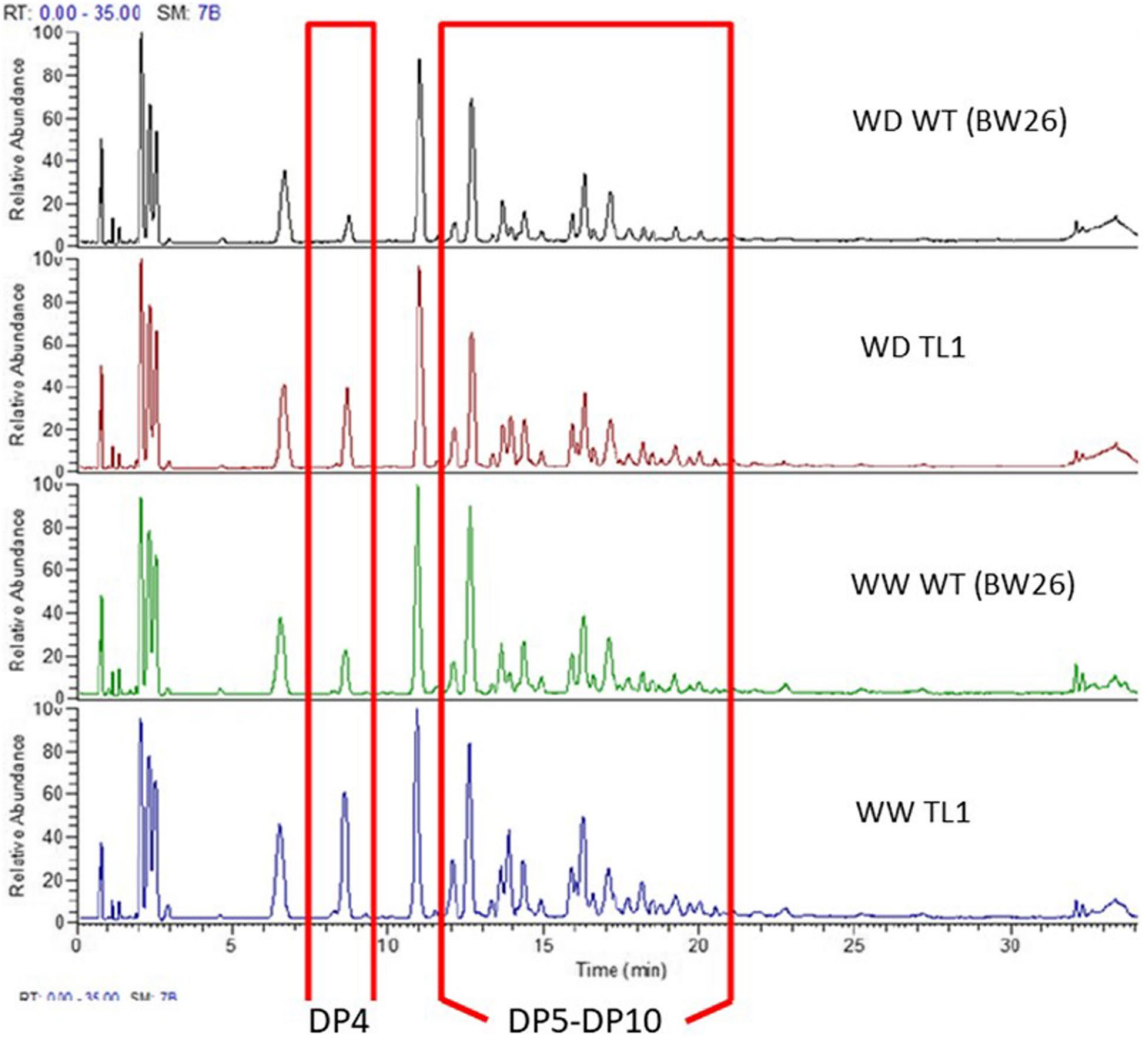
Example of LC–MS chromatograph showing different DP profiles, especially DP4, between TL1 and WT (BW26) grain samples from WW and WD conditions

The analysis of leaf samples showed that in all WW plants across genotypes, a similar level of DP3 fructan polymers was detected (Fig. 4a). WW transgenic plants from events TL1 and TL2 respectively showed approximately twofold and 1.5-fold increase in high-DP fructan polymers (DP > 4) compared to NT plants (Fig. 4a). In general, WD leaf-sampled plants showed at least a 50% reduction in their fructan level compared to WW plants (Fig. 4a). In particular, event TL1 showed a twofold increase in ≥ DP5 polymers compared to NT plants. WD leaf samples showed similar levels of fructan polymers among event TL2, and NT plants (Fig. 4a). The analysis of stem samples showed that in all WW plants across lines, a similar level of fructan was detected and the dominant fructan polymers were DP5, DP6 and DP7 (Fig. 4b). The comparison of WW to WD non-transgenic plants, showed a 70% reduction in high-DP fructan polymers, DP5, DP6 and DP7 (Fig. 4b). With WD treated plants, the transgenic events TL1 and TL2, showed a 1–twofold increase in DP4 fructan polymers compared to NT lines (Fig. 4b). The analysis of grain samples between transgenic Event TL2 and NT lines from WW plants, showed similar levels of fructan polymers from DP3 to DP10 (Fig. 4c). With WW plants, grain from only transgenic event TL1 showed a significant increase in DP4 and higher fructan polymers compared to non-transgenic control lines (Figs. 4c and 5). Difference in DP3 fructan polymer level was not observed between event TL1 and non-transgenic grain (Fig. 4c). In WD grain samples, all lines showed similar levels of fructan accumulation across polymers DP3 to DP10 (Fig. 4c).

### Grain analysis

The quantitative comparison between fructans of different DP values and measurement of grain sucrose and total fructan content was carried out with grain samples from transgenic events TL1 and TL2 and non-transgenic WT and null-sibling plants (Table 1). LC–MS based WSC analysis in grain samples from events TL1 and TL2 showed less (15–20%) sucrose accumulation compared with WT plants under WW and WD conditions. The water condition did not impact sucrose accumulation. In contrast, the water deficit treatment effected fructan and total WSC composition (Supp. Table 4). Under both WW and WD conditions, an increase in grain fructan accumulation was found only with event TL1. With event TL1, grain samples showed a shift of the fructan-sucrose ratio by 50% and 40% from WW and WD conditions respectively. With event TL2, a 30% increase in fructan-sucrose ratio was observed with the WD but not with the WW condition. No significant difference in total grain WSC concentration was measured between transgenic and WT samples with only a minor increase (10%) in event TL1 under both WW and WD conditions (Table 1).

**Table 1:** The grain WSC composition of WT, null and transgenic lines from WW and WD conditions

**Grain component (% Dry Grain Weight)**	**Water condition**	**WT**	**Null**	**TL1**	**TL2**
**Sucrose**	WW	0.80 ± 0.07^b^	0.70 ± 0.05^ab^	0.65 ± 0.04^a^	0.63 ± 0.11^a^
	WD	0.73 ± 0.06^b^	0.76 ± 0.08^ab^	0.63 ± 0.05^a^	0.63 ± 0.06^a^
	Combined	0.77 ± 0.07^b^	0.73 ± 0.07^ab^	0.64 ± 0.04^a^	0.63 ± 0.08^a^
**Fructan**	WW	1.64 ± 0.16^ab^	1.65 ± 0.05^ab^	2.05 ± 0.24^b^	1.58 ± 0.17^a^
	WD	1.34 ± 0.15^a^	1.44 ± 0.05^a^	1.62 ± 0.16^a^	1.48 ± 0.07^a^
	Combined	1.49 ± 0.22^a^	1.55 ± 0.13^a^	1.84 ± 0.30^b^	1.53 ± 0.13^a^
**Total WSC**	WW	2.45 ± 0.10^ab^	2.35 ± 0.08^ab^	2.71 ± 0.23^b^	2.21 ± 0.27^a^
	WD	2.06 ± 0.19^a^	2.19 ± 0.02^a^	2.25 ± 0.14^a^	2.11 ± 0.09^a^
	Combined	2.26 ± 0.25^ab^	2.27 ± 0.10^ab^	2.48 ± 0.30^b^	2.16 ± 0.19^a^

Values are Mean ± standard deviation (*n* = 3). Within each grain component, statistical significance using Tukey’s 95% confidence intervals was determined between means for (indicated by superscript letters): Line (sucrose, fructan, total WSC); Line x Water Condition (fructan and total WSC); and combined water condition treatment groups, see supp. Table 4 for ANOVA tables

## Discussion

Transgenic events TL1 and TL2 were selected in this study through a segregation evaluation at T1, T2 and T3 generations among a total of 20 independent transgenic events (Supplementary Table 1). At T1 and T2 generations, PCR-based segregation and transgene copy-number analysis was effective in determining that 9 of 20 transgenic events contained a homozygous single-locus transgene integration. We considered that stably-inherited, single-copy, homozygous plants to be important for transgene transcriptional analysis to exclude dosage effects of multiple transgene integrations and the potential of unstable expression across generations [36–39]. The transgene zygosity was an important factor since the aim of this study was to enhance the activity of the endogenous fructan biosynthesis pathway, thus transgene expression levels and metabolic products required precise quantitative measurements that could be confounded by hemizygosity and genetic segregation. For comparative analysis, we included null azygous segregants (or null-sibling lines) to mitigate the risk of somaclonal variation that could confound a transgene-induced phenotype [40]. In our study, the null line was shown with a normal and consistent phenotype, WSC accumulation and yield compared to WT plants under both WW and WD conditions. No observable somaclonal variation or aberrant effect of transformation was found and the observed differences between transgenic and NT plants appeared to be indeed due to transgene functions.

The transcriptional fusion between *Ta1SST* and *Ta6SFT* gene open reading frames (ORFs) was regulated by the highly-active and green tissue-specific *RbcS* promoter from wheat [19]. Therefore, the overexpression of this transgene was expected to occur in major photosynthetic tissues, such as leaves [41], and the transcription was confirmed with in transgenic leaves using RT-ddPCR. In our observations, transgenic plants showed similar levels of endogenous expression of genomic fructan biosynthesis pathway genes as WT plants under both WW and WD conditions (Fig. 2B), which indicated that no transgene-induced effect can be observed on endogenous fructan biosynthesis expression. We also observed that WD treatments can reduce the transcriptional activities of fructan biosynthesis pathway genes, including both transgenic and endogenous genes. Theoretically, such suppression on fructan biosynthesis genes would reduce the enzyme activities involved in fructan synthesis, and affect the balance between fructan biosynthesis and degradation in plants. Similar observations have been reported where increased fructan exohydrolases enzyme activities and reduced fructan biosynthesis enzyme activities in wheat plants under drought stress conditions [24].

The level of transcription was shown to vary between events, with TL1 considerably higher than TL2 (Fig. 2) and reduced with plant maturity [42]. These levels of transgene expression corresponded with *in-vivo* fructan accumulation with both WW and WD-treated plants (Fig. 3). However, as TLC based WSC analysis includes other non-fructan carbohydrate polymers, sub-grouping of fructan polymers in LC–MS analysis based on DP values was conducted from leaf, stem and grain samples (Fig. 4). Increased high-DP fructan polymers were shown to accumulate in transgenic plants, particularly in event TL1 leaf and stem samples. The difference in fructan expression levels, thus fructan accumulation between transgenic events TL1 and TL2, may be attributed to genome position due to the nature of random transgene integration [43–45]. Since only a small number of events were characterised, it is prudent to acknowledge that this was a major limitation in this study, whereby the potential to optimise and validate high fructan events would be required through further development and screening of transgenic events or utilising precise site-directed genome editing technologies [45].

Drought or WD, is a major constraint on yield in dryland farming systems in Australia, with the greatest impact observed during anthesis and grain-fill [46–48]. Thus, in this greenhouse study, WD conditions were induced at 15 days post-anthesis to simulate stress at a critical stage for grain development and yield. Fructans can serve as an indirect source of hexose sugars during degradation to maintain osmotic potential and stabilise cell membranes [6]. In contrast, similar levels of stem fructan accumulation were observed among transgenic and non-transgenic plants when grown under WW conditions.

To obtain a substantial increase in accumulation of high-DP fructan polymers in wheat plants, overexpression of two fructan biosynthesis pathway genes (*Ta1SST:Ta6SFT*) was found to be beneficial. Overexpression of the *Ta1SST* gene alone was not sufficient to induce increased high-DP fructan polymer accumulation [42]. Our observation was in accordance with a tobacco transformation study, in which plants were transformed with either single or combinations of *Ta1SST*, *Ta1FFT* and *Ta6SFT* genes, and transformed plants containing both *Ta1SST* and *Ta6SFT* were shown to have the most fructan biosynthesis activity and accumulation [34].

In general, fructans were mainly stored as DP3 and DP4 polymer in wheat grain under both WW and WD conditions (Fig. 4c). Overall, fructan accumulation levels between transgenic and non-transgenic grain were not remarkably different as this accounted for the presence of all polymers (Table 1). However, LC–MS analysis did show that transgenic grain (TL1) contained a higher level of polymers, such as DP4 and DP5 (Figs. 4c and 5). The increase in grain fructan polymers such as, DP4 and DP5, was probably due to fructan remobilisation from vegetative tissues rather than from in situ synthesis during grain development [3, 18–21]. Under drought conditions, exohydrolases, such as of 1- fructan exohydrolases (1-FEH), have a major role in fructan remobilisation from stems during grain fill [24, 49]. Therefore, with transgenic plants that overexpress fructan biosynthesis genes, it was possible that FEHs could up-regulate in response, which warrants further investigation into optimising network interactions for improved fructan metabolism.

## Conclusions

This study has shown transgenic plants with increased accumulation of high DP fructan polymers due to transformation with a construct that overexpressed *Ta1SST* and *Ta6SFT* fusion genes. Despite the major limitation of presenting less than three events, this study showed the potential to develop transgenic wheat plants with elevated fructan levels, which can be a value-adding trait to wheat and grain quality. Effective deployment of high fructan GM wheat, would require further work for optimal expression and validation under various conditions for improved stress tolerance.

## Methods

### Transformation vectors

The coding DNA sequences of *Ta1SST* and *Ta6SFT* [13] were designed as one transcriptional fusion sequence in silico (Vector NTI 11, Thermo Fisher Scientific, USA). This fusion sequence was regulated by a *TaRbcS* promoter and terminator [19] (Fig. 1a). The transgene sequence (*TaRbcS-p_Ta1SST:Ta6SFT*_*TaRbcS-t*) was synthesized using the Invitrogen™ GeneArt™ Gene Synthesis service (Thermo Fisher Scientific, USA), and was then transferred into the transfer DNA (T-DNA) region of a binary expression vector using GATEWAY® recombination cloning technology (Invitrogen, USA). The T-DNA region of the binary vector also contained a selectable marker cassette (*OsActin-p_bar_CaMV35s-t*) that comprised a synthetically constructed *bar* gene [50], that encoded for phosphinothricin acetyltransferase (PAT), and regulatory sequences from the rice actin promoter and Cauliflower Mosaic Virus Terminator (Fig. 1b). The final T-DNA vectors were confirmed using Sanger sequencing.

### Generation of transgenic events

Transgenic wheat plants, *Triticum aestivum* cv. Bobwhite26RH, were generated via *Agrobacterium*-mediated transformation using immature zygotic embryos as an explant source. Initially, 20 independent T0 transgenic in vitro events were selected and acclimatised in greenhouse conditions for molecular analysis and seed production. In T0 transgenic plants, qPCR detected the presence and transgene copy number of the *OsActin1* promoter element in all 20 events. All 20 events were maintained and self-pollinated from T1 to T4 generation. Selection of homozygous single-locus events was achieved by qPCR detection and copy number of the transgene element and the segregating transgene locus and chi-square test with at least 12 sibling plants at the T1 and T2 generations. Nine events with appropriate segregation were further analysed and confirmed at the T3 generation, as transgene single-locus homozygous events that contain 2 copies of the transgene. Of these, two events were further selected based on the presence of the transgene across all siblings tested and increased detection of WSC accumulation using TLC in leaf and stem samples under WD conditions. Two independent transgenic events (TL1 and TL2) were selected to represent high and moderate WSC accumulating events, respectively. T4 plants from TL1 and TL2 were then used for further assessment (Supplementary Table 1). In this study, non-transgenic plants will refer to both sibling nulls derived from transgenic events and non-transformed wild-type plants (WT).

### Greenhouse growth and treatment conditions

Transgenic plants (T4 generation) from TL1 and TL2, non-transgenic (WT and null) plants were grown in a greenhouse with 16/8 h photoperiod, 26 °C/16 °C ± 2˚C day /night temperature; and supplemented with 350 µmol m^−2^ s^−1^ photosynthetic photon flux density from high pressure sodium growth lights. All wheat plants were grown in conventional potting media based on composted pine bark (Van Schaik’s Bio Gro, Australia) supplemented with 16.67 mL.L^−1^ coarse vermiculite, 11.11 mL.L^−1^ coarse perlite, 1.11 g.L^−1^ of large nutricote, 0.50 g.L^−1^ of trace elements, 0.22 g.L^−1^ lime and 0.94 g.L^−1^ nitrogen (Fertool, Australia).

A complete randomized design was used with four lines and were divided into two treatment groups, well-watered (WW) and water-deficit (WD). Three plants from each line of each treatment were grown in a 200 mm pot and were treated as one experimental unit (EU). In total, 12 plants from 4 EUs per line per treatment were assessed. The WD treatment was induced by ceasing irrigation at anthesis (at approximately 60 day-post-sowing, dps) for a duration of 15-days (Supplementary Fig. 2). The media moisture level was monitored daily and was recorded as soil-gravimetric water content (SGWC). All WD treated plants experienced two-cycles of WD treatment with SGWC lowered to approximately 15% at 65 dps and 74 dps. After each cycle of WD treatment, plants were re-watered to 100% SGWC. SGWC of WW plants were maintained above 40%.

For transcription and metabolite (WSC) analysis, samples from both WW and WD plants of each line and treatment at 75 dps were collected and snap frozen between 12:00–14:00 EST on the day of sampling. Grain samples were harvested from plants of each line and treatment at full maturity for metabolite analysis.

### Transcription analysis

Total RNA was extracted from 0.5 g of leaf tissue from the primary tiller and treated with DNase using a Qiagen® RNeasy Mini Kit following the manufacturer’s protocol. cDNA was synthesised from total RNA using the iScript™ Select cDNA Synthesis Kit with random primer mix (Bio-Rad Laboratories, USA) following manufacturer’s instructions. Both extracted RNA and constructed cDNA samples were checked with the Nanodrop™ 2000 spectrophotometer (Thermo Fisher Scientific, USA), for yield and quality based absorbance at A260/A280 nm.

Transgene expression analysis was conducted on the cDNA samples using a QX200™ Droplet Digital™ PCR System (DD-PCR) (Bio-Rad Laboratories, Inc., USA). Each biological sample was tested with four technical replicates in three runs using specific designed primers and probes using online IDT PrimerQuest Tool (www.idtdna.com) (Table 2). Primers and probes were designed to amplify (i) the unique fragment of the fusion site between two fructan biosynthesis genes (*Ta1SST:Ta6SFT*) for transgene-specific expression; (ii) the three-prime untranslated region (3’-UTR) of endogenous *Ta1SST* gene for genomic *Ta1SST*-specific expression; and (iii) the coding sequence (CDS) of *Ta1SST* gene for overall *Ta1SST* expression. All relative gene expression analysis was normalised against an endogenous reference gene *Ta2776* [35]. Relative expression level was also validated using the normalization of transgene-specific expression against a second reference gene, *TaGAPDH* [51] (data not shown).

**Table 2:** Primers and probes used in dd-PCR

**Targeted allele**	**Type**	**Sequence**
*Ta1SST:Ta6SFT fusion region*	*forward primer*	*5’-GCATGATATGGACTCGTCGTA-3’*
	*reverse primer*	*5’-GAGGGCAGTGGCTTGTA-3’*
	*probe (FAM)*	*5’-TGACTTGGTAGTCGTCGATGGGTCA-3’*
*Ta1SST gene coding region*	*forward primer*	*5’-TACGCGTCCAAGTCCTTCTA-3’*
	*reverse primer*	*5’-AGGTTCGTCCGGGTCTT-3’*
	*probe (FAM)*	*5’-CGATTCCGAGGACAGTGGAGCTTG-3’*
*Ta1SST gene 3’UTR region*	*forward primer*	*5’-GCATGATATGGACTCGTCGTA-3’*
	*reverse primer*	*5’-CCGAGAACAACCGATCCATAAC-3’*
	*probe (FAM)*	*5’-ACACAGATGATGACTTGGTAGTCGTCGA-3’*
*Ta2776 gene coding region*	*forward primer*	*5’-CAAGTACCCTACCATGAGCAAA-3’*
	*reverse primer*	*5’-CTCACCAAGCATCACAACAATC-3’*
	*probe (HEX)*	*5’-TCAAGCTCTCTGTTGTTGAGGGTGA-3’*
*TaGAPDH gene coding region*	*forward primer*	*5’-GTGTTCCCACTGTTGATGTTTC-3’*
	*reverse primer*	*5’-CCTCCTTGATAGCAGCCTTAAT-3’*
	*probe (HEX)*	*5’-AGACTTGCGAAGCCAGCAACCTAT-3’*

PCR thermocycling conditions comprised one cycle of enzyme activation at 95 °C for 10 min, then 40 cycles of denaturation and annealing at 94 °C and 60 °C for 30 s and 1 min, respectively, and final cycle for enzyme deactivation at 98 °C for 10 min. The heated lid was set to 105 °C and total reaction volume was 40 µL according to manufacturer’s kit instructions (ddPCR™ Supermix for Probes, Bio-Rad Laboratories, USA). The PCR results were validated and analysed using the QuantaSoft™ Analysis Pro Software (Bio-Rad Laboratories, Inc., USA).

### Total WSC extraction

The samples from the flag leaf and first above ground internode (stem) of the primary tiller and grain were freeze-dried for 2 days (pressure < 200 × 10^–3^*mBar*; temperature < -40˚C) and were then ground into fine powder using 2010 Geno/Grinder® (SPEX SamplePrep, USA). The ground tissue powder was then incubated in water with a heater block at 90 °C for 60 min to extract the total WSC [52].

### Thin Layer Chromatography (TLC)

TLC analysis was conducted to detect and semi-quantify total WSC accumulation in extractions from leaf and stem samples. WSC extract (2 µL) from each sample was loaded into TLC plates and compared to 2 µL of 2 mg/mL reference analytes of sucrose, fructose and inulin (Sigma-Aldrich, USA). The TLC plates were developed in the mobile phase solvent containing 45% v/v 1-propanol, 35% v/v ethyl acetate and 20% v/v water for 4 h at room temperature. The WSCs were visualized by spraying the plate with urea-phosphoric acid and followed by incubation at 110 °C for 10 min.

### Liquid Chromatography—Mass Spectrophotometry (LC–MS)

LC–MS was used for comparing the relative abundance of fructan polymers of differing DP in the extracts of leaf, stem and grain samples. Separations of fructan polymers were achieved by using a Thermo Fisher Scientific® Hypercarb™ column (100 × 2.1 mm, 5 µm). The mobile phase was composed of water containing 0.1% formic acid (A) and acetonitrile containing 0.1% formic acid (B). The gradient elution was performed at a flow rate of 0.3 ml/min with the following programme: initial 5% B hold for 5 min, to 20% B after 30 min, to 60% B at 32 min, then return to 5% B at 33 min for a total run time of 40 min. Analyte detection was by Orbitrap mass spectrometer (Thermo Fisher Scientific, USA) with electrospray ionization in negative mode. The heated capillary was maintained at 300 °C with a source heater temperature of 350 °C. The sheath, auxiliary, and sweep gases were at 40, 15 and 8 units respectively. Source voltage was set to 3.2 kV for negative mode. Data were collected over the mass range of 300–2,000 m*/z* at 60,000 resolution. The data were processed using Xcalibur™ software provided by the manufacturer. Due to a lack of available standards, relative abundances were used to compare the accumulation level of different fructan polymers between samples (Supplementary Fig. 2).

### High Performance Liquid Chromatography (HPLC)

Total fructan quantification in ground grain samples was performed using high performance anion exchange chromatography (HPAEC) analysis. An aliquot of the extract was hydrolyzed using a mild thermal-acid hydrolysis method (30 mM HCl in a heating block at 70 °C for 90 min). The total fructan level can then be calculated based on the released fructose and glucose molecules following hydrolysis [52, 53]. WSC analysis was performed using an ICS-5000 HPLC system (Dionex) equipped with a CarboPac™ PA100 column (4 × 250 mm) and an electrochemical detector working in a pulsed amperometric mode using a gold working electrode and a combined pH-Ag/AgCl reference electrode. A standard quadruple-potential waveform for carbohydrates was used for all analyses (Supplementary Table 2). The elution program used for quantification of fructose, glucose, sucrose and raffinose is shown in Supplementary Table 3.

### Statistical analysis

Grain WSC components were analyzed with two-way ANOVA and Tukey’s 95% confidence intervals for post-hoc multiple comparisons of means using Genstat (22nd Edition). Residual values were graphically examined to ensure normality and homogeneity of variances.

### Supplementary Information

**Supplementary Material 1.**

## Data Availability

All supporting data for this manuscript are included in additional files. Further enquiries regarding data availability and materials can be directed to the corresponding author.

## Abbreviations

Ta1SST: Wheat sucrose: sucrose 1-fructosyltransferase
Ta6SFT: Wheat sucrose: fructan 6-fructosyltransferase
TaRbcS: Wheat ribulose-1,5-bisphosphate carboxylase/oxygenase small subunit
WD: Water-deficit
DP: Degree of polymerisation
NT: Non-transgenic
WSC: Water-soluble carbohydrates
1-SST: Sucrose: sucrose 1-fructosyltransferase
1-FFT: Fructan: fructan 1-fructosyltransferase
6-SFT: Sucrose: fructan 6-fructosyltransferase
6G-FFT: Fructan: fructan 6G-fructosyltransferase
ROS: Reactive oxygen species
WW: Well-watered
WT: Wild type
TLC: Thin Layer Chromatographic
LC-MS: Liquid Chromatography—Mass Spectrophotometry
PCR: Polymerase chain reaction
GM: Genetically modified
ORFs: Open reading frames
1-FEH: 1- Fructan exohydrolases
T-DNA: Transfer DNA
PAT: Phosphinothricin acetyltransferase
EU: Experimental unit
SGWC: Soil-gravimetric water content
3’-UTR: Three-prime untranslated region
CDS: Coding sequence
HPLC: High Performance Liquid Chromatography
HPAEC: High performance anion exchange chromatography
