# Supplementary material for: Modified fructan accumulation through overexpression of wheat fructan biosynthesis pathway fusion genes Ta1SST:Ta6SFT
Source: BMC Plant Biol. 2024 Apr 30;24:352. doi: 10.1186/s12870-024-05049-w (PMC11059666; doi:10.1186/s12870-024-05049-w)
Supplement: Supplementary file 1 — Supplementary Material 1. [file 12870_2024_5049_MOESM1_ESM.docx]

Chen et al

# Supplementary Tables and Figures

Supplementary Table 1. Event selection process of 20 events from T1 to T3 generations for single locus, transgene homozygous events with increased WDS tolerance.

Supplementary Table 2. Quadruple potential waveform used in HPAEC-PAD analysis for carbohydrate.

Supplementary Table 3. Gradient elution programme used for the quantification of glucose, fructose, sucrose, and raffinose.

*Supplementary Table 4. ANOVA tables for grain WSC components*

Variate: Sucrose

Source of variation d.f. s.s. m.s. v.r. F pr.

Line 3 0.080313 0.026771 6.12 0.006

Trt 1 0.000924 0.000924 0.21 0.652

Line.Trt 3 0.013809 0.004603 1.05 0.396

Residual 16 0.069935 0.004371

Total 23 0.164981

Variate: Fructan

Source of variation d.f. s.s. m.s. v.r. F pr.

Line 3 0.45613 0.15204 7.07 0.003

Trt 1 0.41539 0.41539 19.32 <.001

Line.Trt 3 0.09277 0.03092 1.44 0.269

Residual 16 0.34401 0.02150

Total 23 1.30831

Variate: Total_WSC

Source of variation d.f. s.s. m.s. v.r. F pr.

Line 3 0.32155 0.10718 4.10 0.025

Trt 1 0.45550 0.45550 17.42 <.001

Line.Trt 3 0.13414 0.04471 1.71 0.205

Residual 16 0.41835 0.02615

Total 23 1.32954


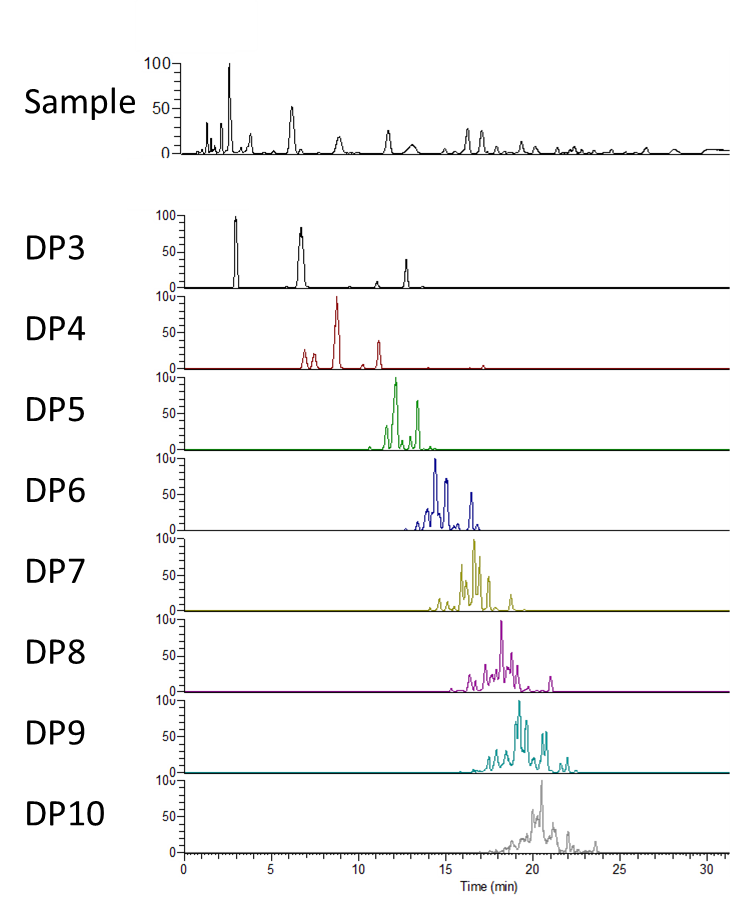


*Supplementary Figure 1. Extracted Ion Chromatography (EIC) of fructan polymers from DP3 to DP10 in wheat leaf samples.*


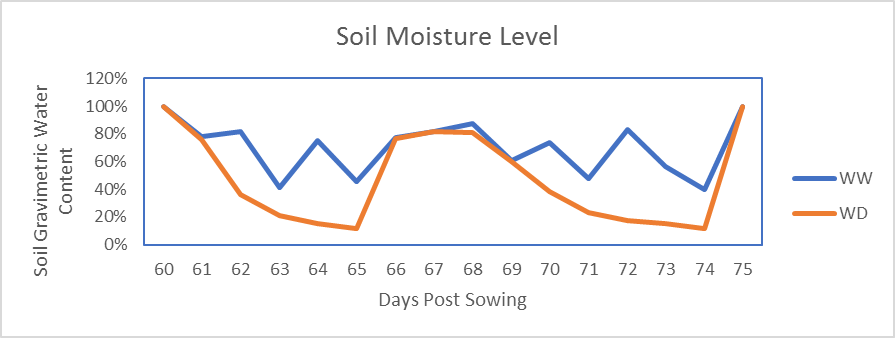


*Supplementary Figure 2. Soil Moisture level of WW and WDS treated plants from 60 to 75 days post sowing.*
